# Low plasma PD-L1 levels, early tumor onset and absence of peritoneal carcinomatosis improve prognosis of women with advanced high-grade serous ovarian cancer

**DOI:** 10.1186/s12885-023-10911-5

**Published:** 2023-05-13

**Authors:** Daniele Fanale, Chiara Brando, Lidia Rita Corsini, Sofia Cutaia, Mariano Catello Di Donna, Ugo Randazzo, Clarissa Filorizzo, Chiara Lisanti, Luigi Magrin, Vittorio Gurrera, Raffaella Romano, Alessandra Dimino, Tancredi Didier Bazan Russo, Daniel Olive, Salvatore Vieni, Gianni Pantuso, Antonio Giordano, Vito Chiantera, Antonio Russo, Viviana Bazan, Juan Lucio Iovanna

**Affiliations:** 1 Section of Medical Oncology, Department of Surgical, Oncological and Oral Sciences, University of Palermo, Via del Vespro 129, Palermo, 90127 Italy; 2 Department of Gynecologic Oncology, University of Palermo, Palermo, 90127 Italy; 3 Medicine and Surgery School, University of Palermo, Palermo, 90127 Italy; 4 Team Immunity and Cancer, Centre de Recherche en Cancérologie de Marseille (CRCM), INSERM U1068, CNRS UMR 7258, Aix-Marseille Université and Institut Paoli-Calmettes, Marseille, France; 5 Division of General and Oncological Surgery, Department of Surgical, Oncological and Oral Sciences, University of Palermo, Palermo, 90127 Italy; 6 Sbarro Institute for Cancer Research and Molecular Medicine and Center of Biotechnology, College of Science and Technology, Temple University, PA Philadelphia, 19122 USA; 7 Department of Biomedicine, Neuroscience and Advanced Diagnostics, University of Palermo, Palermo, 90127 Italy; 8 Centre de Recherche en Cancérologie de Marseille (CRCM), INSERM U1068, CNRS UMR 7258, Aix-Marseille Université and Institut Paoli-Calmettes, Parc Scientifique Et Technologique de Luminy, Marseille, 13288 France

**Keywords:** BTLA, BTN2A1, BTN3A1, Butyrophilins, HGSOC, Immune checkpoints, PD-1, PD-L1, Prognostic biomarkers

## Abstract

**Background:**

The most common subtype of ovarian cancer (OC) showing immunogenic potential is represented by the high-grade serous ovarian cancer (HGSOC), which is characterized by the presence of tumor-infiltrating immune cells able to modulate immune response. Because several studies showed a close correlation between OC patient’s clinical outcome and expression of programmed cell death protein-1 or its ligand (PD-1/PD-L1), the aim of our study was to investigate if plasma levels of immunomodulatory proteins may predict prognosis of advanced HGSOC women.

**Patients and methods:**

Through specific ELISA tests, we analyzed plasma concentrations of PD-L1, PD-1, butyrophilin sub-family 3A/CD277 receptor (BTN3A1), pan-BTN3As, butyrophilin sub-family 2 member A1 (BTN2A1), and B- and T-lymphocyte attenuator (BTLA) in one hundred patients affected by advanced HGSOC, before surgery and therapy. The Kaplan–Meier method was used to generate the survival curves, while univariate and multivariate analysis were performed using Cox proportional hazard regression models.

**Results:**

For each analyzed circulating biomarker, advanced HGSOC women were discriminated based on long (≥ 30 months) *versus* short progression-free survival (PFS < 30 months). The concentration *cut-offs*, obtained by receiver operating characteristic (ROC) analysis, allowed to observe that poor clinical outcome and median PFS ranging between 6 and 16 months were associated with higher baseline levels of PD-L1 (> 0.42 ng/mL), PD-1 (> 2.48 ng/mL), BTN3A1 (> 4.75 ng/mL), pan-BTN3As (> 13.06 ng/mL), BTN2A1 (> 5.59 ng/mL) and BTLA (> 2.78 ng/mL). Furthermore, a lower median PFS was associated with peritoneal carcinomatosis, age at diagnosis > 60 years or Body Mass Index (BMI) > 25. A multivariate analysis also suggested that plasma concentrations of PD-L1 ≤ 0.42 ng/mL (HR: 2.23; 95% CI: 1.34 to 3.73; *p* = 0.002), age at diagnosis ≤ 60 years (HR: 1.70; 95% CI: 1.07 to 2.70; *p* = 0.024) and absence of peritoneal carcinomatosis (HR: 1.87; 95% CI: 1.23 to 2.85; *p* = 0.003) were significant prognostic marker for a longer PFS in advanced HGSOC patients.

**Conclusions:**

The identification of high-risk HGSOC women could be improved through determination of the plasma PD-L1, PD-1, BTN3A1, pan-BTN3As, BTN2A1 and BTLA levels.

**Supplementary Information:**

The online version contains supplementary material available at 10.1186/s12885-023-10911-5.

## Introduction

Ovarian cancer (OC) is the seventh most commonly diagnosed cancer and the eighth leading cause of cancer death in women worldwide, with a 5-year relative survival of 49% [1].

Among epithelial OCs, which represent the most common subtype, high-grade serous ovarian cancer (HGSOC) is the most frequent and is responsible for 70–80% of all OC deaths [2].

High heterogeneity and resistance to therapy significantly contribute to the poor prognosis of HGSOC. Surgery and platinum-based chemotherapy are the standard treatment in OC [3]. Alternatively, neoadjuvant chemotherapy followed by interval debulking surgery has been shown to improve progression-free survival (PFS) and overall survival (OS) [2]. Despite this, recurrence rate still remains high and about 70% of women with advanced OC relapses with a poor prognosis [4]. Immunotherapy, which has already proved to be effective in other tumors, such as melanoma [5], renal cell carcinoma [6, 7], and non small cell lung cancer [8], has also attracted attention in OC based on the finding that many OCs have tumor-infiltrating lymphocytes (TILs) [9]. However, the use of drugs directed against immune receptors and their ligands, so-called immune checkpoint inhibitors (ICIs), has not produced the expected results in OC [10]. ICIs act by blocking immune checkpoints, the “brakes” of the immune system, which under physiological conditions mediate self-tolerance and modulate the duration and magnitude of physiological immune responses [11]. However, tumor cells express high levels of inhibitory immune signaling proteins, exploiting them to inactivate TILs and escape from immune surveillance [12].

One of the most studied immune checkpoint receptors is the programmed cell death protein 1 (PD-1), with its ligands, PD-L1 and PD-L2, which are involved in the activation, proliferation and cytotoxic secretion of T cells [9]. PD-L1 expression in tumor tissue correlates with the response to ICIs in different solid tumors, including Non-Small-Cell Lung Cancer (NSCLC) [8], and endometrial [13], triple-negative breast [14], head and neck tumors [15]. proving to be a useful biomarker [10]. Although more than 50% of advanced OCs expresses PD-L1, early-phase clinical trials on efficacy of anti-PD-1/PD-L1 agents showed an overall response rate (ORR) between 8–60% and a median PFS of 2–10 months [16].

Other immune checkpoints involved in the interaction between cancer cells and T lymphocytes showed an interesting immunomodulatory role in different tumors [6]. Among these, transmembrane glycoproteins belonging to the immunoglobulin superfamily, called butyrophilins (BTNs), such as butyrophilin sub-family 3A/CD277 receptors (BTN3A) sub-family, including BTN3A1 and pan-BTN3A, butyrophilin sub-family 2 member A1 (BTN2A1), and the B and T lymphocyte attenuator (BTLA) belonging to the B7-like receptors, could represent novel target immune checkpoints [17].

In order to investigate the potential prognostic role of these immune checkpoints in OC, we assessed whether circulating soluble forms of PD-L1 (sPD-L1), PD-1 (sPD-1), BTN3A1 (sBTN3A1), pan-BTN3As (pan-sBTN3As), BTN2A1 (sBTN2A1) and BTLA (sBTLA) may be useful to predict prognosis in advanced HGSOC patients.

## Materials and methods

### Study population

A prospective analysis was performed on a cohort of one hundred patients with advanced-stage HGSOC recruited at the two Sicilian hospital centers: “Sicilian Regional Center for the Prevention, Diagnosis and Treatment of Rare and Heredo-Familial Tumors” of the Section of Medical Oncology of University Hospital Policlinico “P. Giaccone” of Palermo (Italy), and Department of Gynecologic Oncology of the Hospital ARNAS Civico “Di Cristina Benfratelli” of Palermo (Italy). The information concerning the personal history of tumor, age of cancer diagnosis, International Federation of Gynecology and Obstetrics (FIGO) stages, tumor histological subtype and tumor grading were anonymously recorded and coded for all enrolled patients who had previously signed and provided a written informed consent (Table S1). The study (Protocol “TIC-OC v.1.1”) was approved by ethical committee (Comitato Etico Palermo 1) of the University-affiliated Hospital A.O.U.P. (Azienda Ospedaliera Universitaria Policlinico) “P. Giaccone” of Palermo (Italy) [18].

Between May 2018 and July 2021 we prospectively collected blood samples from one hundred women with a confirmed histological diagnosis of advanced HGSOC (stage IIIB-IV) at baseline, before surgery (surgical staging or cytoreductive surgery as clinically indicated) and starting first-line chemotherapy treatment with Carboplatin AUC (area under the curve) 5 and Paclitaxel (175 mg/m2) based on the current therapeutic strategies. In summary, patients were considered eligible for this study based on the previously reported inclusion criteria [18]: i) Histologically confirmed and well documented diagnosis of advanced HGSOC; ii) Women older than 18 years; iii) Availability of peripheral blood from affected patients for the plasma isolation; iv) Therapeutic treatment-naïve patients at the moment of blood sampling. Patients with Eastern Cooperative Oncology Group (ECOG) Performance Status (PS) ≥ 3 were excluded from the study.

In order to confirm the obtained results, another independent cohort of 24 advanced HGSOC patients (validation cohort) recruited at the Section of Medical Oncology of University Hospital Policlinico “P. Giaccone” of Palermo (Italy) was used [18].

### Measurement of plasma PD-L1, PD-1, BTN3A1, pan-BTN3As, BTN2A1, and BTLA concentrations

The peripheral blood samples from untreated patients with advanced HGSOC were collected at baseline, processed for plasma isolation and subsequently stored as previously reported [6, 7, 19].

The plasma sPD-L1, sPD-1, sBTN3A1, pan-sBTN3As, sBTN2A1, and sBTLA concentrations were determined by means of specific enzyme-linked immunosorbent assays (ELISAs). Because other commercially available assays showed some discrepancies, we used specific ELISAs, produced by the company DYNABIO S.A. (Parc de Luminy, Marseille, France), according to the previously reported specifications [18, 20, 21]. Table S2 shows all information regarding the characteristics of six ELISA tests, whose protocol has been previously described [19, 20].

### Statistical analysis

An analysis by ROC curves was performed to determine the optimal concentration *cut-offs* for each soluble form of immune checkpoints and other examined patients characteristics (age at diagnosis and Body Mass Index), in order to discriminate HGSOC patients based on long (≥ 30 months) *versus* short PFS (< 30 months). The Kaplan–Meier method and log-rank test were used to calculate patient PFS. Univariate and multivariate Cox proportional hazard regression models were built to identify significant prognostic factors for PFS [21].

MedCalc software v.18.2.1 for Windows (MedCalc Software, Ostend, Belgium) and GraphPad Prism software v. 9.0.0 (GraphPad Software, San Diego, CA) were used to generate and graphically represent data [21]. *P* values < 0.05 have been considered statistically significant.

## Results

### Stratification of advanced HGSOC patients based on progression-free survival

The plasma concentrations of sPD-L1, sPD-1, sBTN3A1, pan-sBTN3As, sBTN2A1, and sBTLA were measured in peripheral blood from one hundred advanced HGSOC patients, prior to surgery and starting first-line chemotherapy treatment, using specific ELISA tests.

**Fig. 1:**
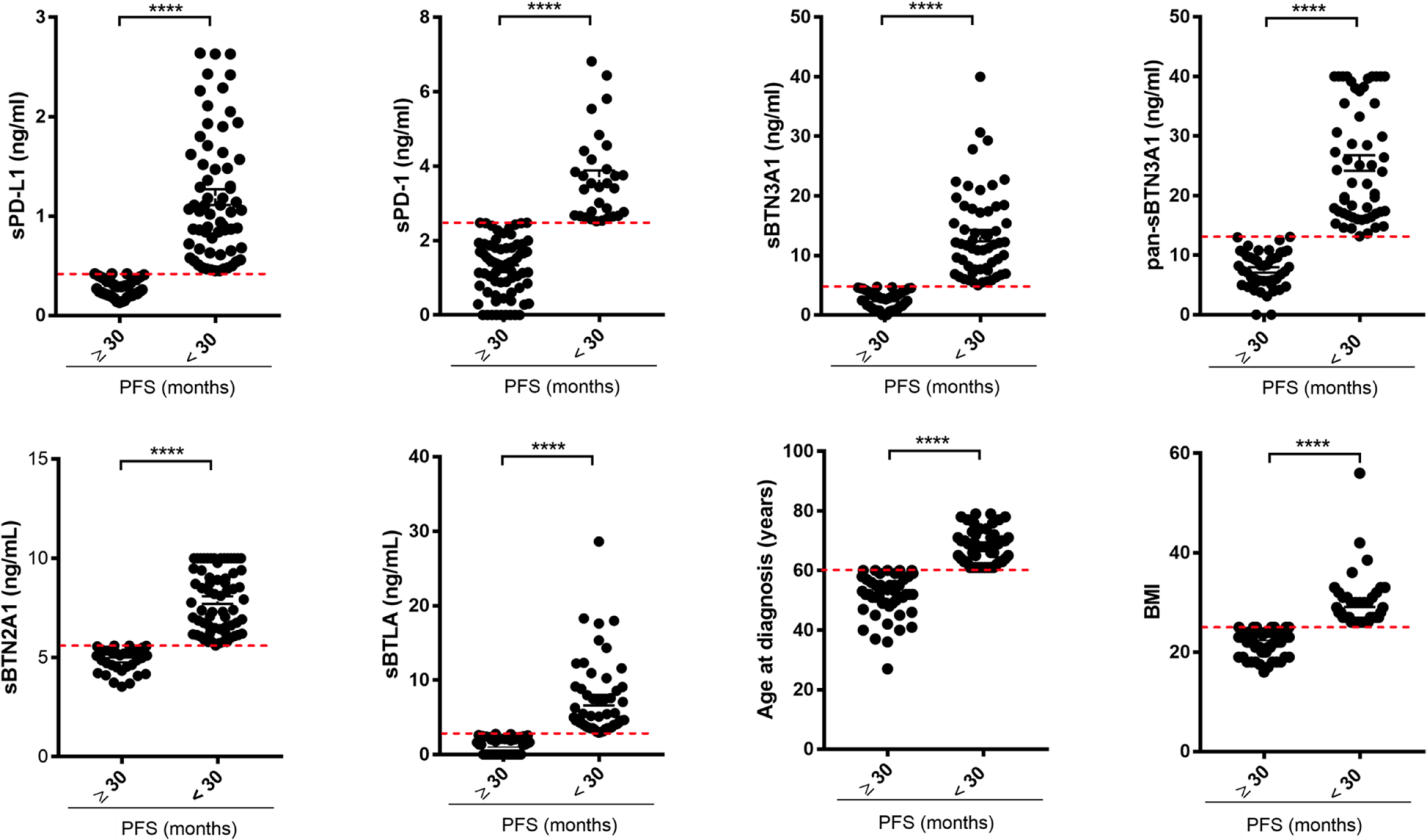
Scatter plots by group discriminating advanced HGSOC patients based on long *versus* short PFS for each examined factor. The plasma levels of each soluble protein, ages at diagnosis and BMIs of advanced HGSOC patients were plotted for short (< 30 months) *versus* long PFS (≥ 30 months). For each considered factor, the red dashed lines represent the optimal thresholds previously calculated by ROC analysis. The concentrations of each biomarker are reported in ng/ml. BMI, Body Mass Index; PFS, Progression-Free Survival. **** = *P* < 0.0001

The receiver operating characteristic (ROC) analysis was carried out to determine for each soluble biomarker the optimal concentration threshold (*Youden index* associated criterion) able to discriminate advanced HGSOC patients based on long (≥ 30 months) *versus* short PFS (< 30 months). At the same time, a further ROC analysis was performed to establish the optimal *cut-offs* of two different parameters: age at diagnosis and BMI. The ROC curve analysis showed that the optimal concentration threshold was 0.42 ng/ml for sPD-L1 (AUC = 0.71, *P* = 0.01), 2.48 ng/ml for sPD-1 (AUC = 0.60, *P* = 0.04), 4.75 ng/ml for sBTN3A1 (AUC = 0.64, *P* = 0.01), 13.06 ng/ml for pan-sBTN3As (AUC = 0.65, *P* = 0.008), 5.59 ng/ml for sBTN2A1 (AUC = 0.64, *P* = 0.02), and 2.78 ng/ml for sBTLA (AUC = 0.62, *P* = 0.02). Furthermore, the best *cut-offs* for age at diagnosis and BMI were 60 years (AUC = 0.67, *P* = 0.002) and 25 (AUC = 0.62, *P* = 0.01), respectively (Figure S1). Interestingly, these thresholds calculated through ROC analysis were close to median concentration values determined for each examined circulating immune checkpoint. Indeed, median concentration values were 0.64 ng/ml for sPD-L1 (range 0.13 to 2.64 ng/ml), 1.80 ng/ml for sPD-1 (range 0 to 6.82 ng/ml), 6.22 ng/ml for sBTN3A1 (range 0 to 40.0 ng/ml), 14.60 ng/ml for pan-sBTN3As (range 0 to 40.0 ng/ml), 6.35 ng/ml for sBTN2A1 (range 3.53 to 10.0 ng/ml), and 3.04 ng/ml for sBTLA (range 0 to 28.61 ng/ml). Also *cut-offs* previously obtained by ROC analysis for age at diagnosis and BMI were close to their median values: 61 years for age at diagnosis (range 27 to 79 years) and 24 for BMI (range 16 to 56). Afterwards, the plasma levels of each molecule, ages at diagnosis and BMIs were graphically represented, in order to discriminate and divide the advanced HGSOC patients into 2 groups at long *versus* short PFS based on each investigated factor (Fig. 1). The red dotted lines were used to indicate the concentration threshold of each circulating biomarker as well as *cut-offs* for age at diagnosis and BMI, previously obtained by ROC analysis. All analyzed factors showed high predictive power. Interestingly, most of advanced HGSOC patients with PFS ≥ 30 months showed lower plasma levels (under specific threshold) for each soluble biomarker (sPD-1, sPD-L1, sBTN3A1, pan-sBTN3As, sBTN2A1, and sBTLA), whereas patients with PFS < 30 months predominantly exhibited higher circulating levels of these molecules. In addition, advanced HGSOC women with PFS less than 30 months were over 60 years of age and had a BMI > 25.

### High plasma levels of sPD-L1, sPD-1, sBTN3A1, pan-sBTN3As, sBTN2A1, and sBTLA negatively correlate with progression-free survival in advanced HGSOC patients

**Fig. 2:**
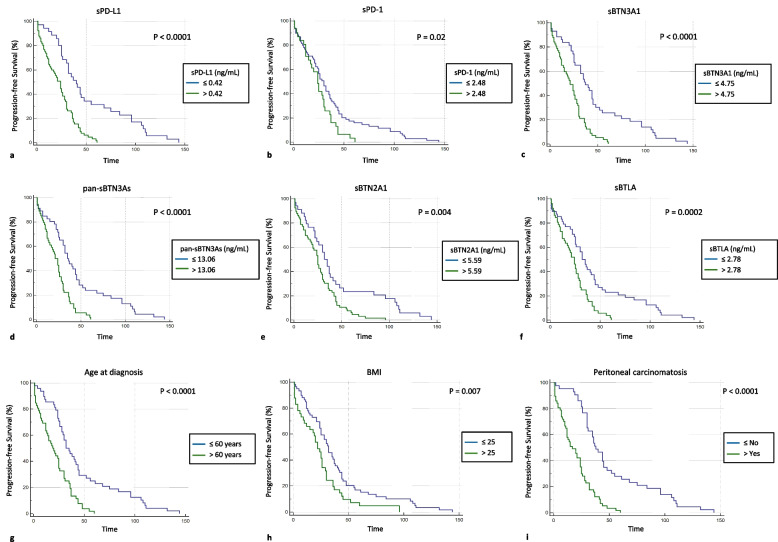
Kaplan–Meier analysis of progression-free survival in one-hundred advanced HGSOC patients with high and low plasma levels of **a** sPD-L1, **b** sPD-1, **c** sBTN3A1, **d** pan-sBTN3As, **e** sBTN2A1 and **f** sBTLA. Also, Kaplan–Meier analyses showing the correlations between PFS and **g** age at diagnosis, **h** BMI or **i** presence of peritoneal carcinomatosis are shown. Abbreviation: BMI, Body Mass Index

Because the clinical impact of circulating immune checkpoints as predictive biomarkers of clinical outcome has yet to be defined in advanced HGSOC patients, a Kaplan–Meier survival analysis was performed to understand the potential prognostic value of circulating sPD-L1, sPD-1, sBTN3A1, pan-sBTN3As, sBTN2A1 and sBTLA concentrations in advanced HGSOC patients, suggesting that their plasma expression levels could be useful, in the future, to predict patient survival. Therefore, we discriminated advanced HGSOC women based on low and high plasma levels for each tested biomarker, using the thresholds previously determined by ROC analysis. Then, the correlation between PFS and circulating levels of the soluble biomarkers was plotted using Kaplan–Meier curves (Fig. 2a-f). For each tested biomarker, patients with plasma levels above and below the specific threshold showed statistically significant differences in PFS. Plasma concentration *cut-offs* associated with poor prognosis and shorter PFS were defined for sPD-L1 (> 0.42 ng/mL), sPD-1 (> 2.48 ng/mL), sBTN3A1 (> 4.75 ng/mL), pan-sBTN3As (> 13.06 ng/mL), sBTN2A1 (> 5.59 ng/mL) and sBTLA (> 2.78 ng/mL) (Fig. 2a-f). Conversely, advanced HGSOC women with plasma concentrations below the indicated thresholds showed a median PFS which was from 6 to 16 months longer than that of subjects with levels above the concentration *cut-offs*. Specifically, patients with high baseline levels of the soluble proteins exhibited the following median PFS values compared to subjects with lower baseline levels: 24 *versus* 40 months for sPD-L1 (95% CI: 14 to 28 *vs* 30 to 55; log-rank *p*-value < 0.0001); 24 *versus* 30 months for sPD-1 (95% CI: 17 to 30 *vs* 24 to 36; log-rank *p*-value = 0.02); 21 *versus* 37 months for sBTN3A1 (95% CI: 15 to 26 *vs* 32 to 45; log-rank *p*-value < 0.0001); 21 *versus* 35 months for pan-sBTN3As (95% CI: 15 to 26 *vs* 30 to 45; log-rank *p*-value < 0.0001); 25 *versus* 32 months for sBTN2A1 (95% CI: 20 to 29 *vs* 24 to 41; log-rank *p*-value = 0.004); and 24 *versus* 32 months for sBTLA (95% CI: 17 to 28 *vs* 25 to 44; log-rank *p*-value = 0.0002). Interestingly, baseline plasma levels of sPD-L1, sBTN3A1 and pan-sBTN3As below their respective *cut-offs* showed a significant benefit in terms of median PFS (14–16 months), whereas a lower advantage in median PFS (6–8 months) was associated with baseline concentrations of sPD-1, sBTN2A1 and sBTLA below their specific thresholds. Therefore, the soluble forms of all proteins investigated in this study have been shown to be potential predictive biomarkers of survival in advanced HGSOC patients.

In addition, we also assessed the impact on PFS of age at diagnosis, baseline BMI and peritoneal carcinomatosis at onset by Kaplan–Meier analysis (Fig. 2g-i). Also for each of these factors, advanced HGSOC women with values above and below the specific thresholds showed statistically significant differences in PFS, suggesting their involvement in predicting prognosis. Poor clinical outcome and shorter PFS were associated with age at diagnosis over 60 years, BMI > 25, or presence of peritoneal carcinomatosis (Fig. 2g-i). In particular, median PFS values for patients with age at diagnosis > 60 years, BMI > 25, or peritoneal carcinomatosis compared to those with features below the respective thresholds were the following: 19 *versus* 32 months for age at diagnosis (95% CI: 13 to 25 *vs* 28 to 44; log-rank *p*-value < 0.0001); 22 *versus* 32 months for BMI (95% CI: 15 to 26 *vs* 25 to 37; log-rank *p*-value = 0.007); and 17 *versus* 38 months for peritoneal carcinomatosis (95% CI: 12 to 24 *vs* 31 to 45; log-rank *p*-value < 0.0001). Interestingly, the better survival advantage was observed in the absence of peritoneal carcinomatosis at the diagnosis.

### Multivariate analysis of several factors associated with PFS in advanced HGSOC patients

Since previous analyses showed that each circulating immune checkpoint (sPD-L1, sPD-1, sBTN3A1, pan-sBTN3As, sBTN2A1, and sBTLA) and other clinic-pathological factors, such as age at diagnosis, baseline BMI and peritoneal carcinomatosis, have a high predictive power of clinical outcome in patients with advanced HGSOC, a multivariate analysis for PFS was performed in order to correlate these variables between them. The prognostic relevance of each biomarker/factor was assessed by an univariate survival analysis using the Cox regression model. The results of the multivariate analysis are reported in Table 1. Age at diagnosis, baseline BMI, peritoneal carcinomatosis at onset, and plasma levels of sPD-1, sPD-L1, sBTN3A1, pan-sBTN3As, sBTN2A1, and sBTLA were observed to be significantly associated with PFS in univariable analyses, whereas in the final multivariable Cox regression model, only the age at diagnosis > 60 years (HR: 1.70; 95% CI: 1.07 to 2.70; *p* = 0.024), presence of peritoneal carcinomatosis (HR: 1.87; 95% CI: 1.23 to 2.85; *p* = 0.003) and plasma levels of sPD-L1 > 0.42 ng/mL (HR: 2.23; 95% CI: 1.34 to 3.73; *p* = 0.002) were statistically significant. No statistically significant association was observed for other considered biomarkers/factors. Therefore, our analysis highlighted that age at diagnosis ≤ 60 years, absence of peritoneal carcinomatosis and expression levels of sPD-L1 ≤ 0.42 ng/mL were independent prognostic factors associated with a longer PFS in patients with advanced HGSOC.

**Table 1:** Univariate and multivariate analysis of biomarkers and other factors for PFS in advanced HGSOC patients

Factor/biomarker	Univariate Cox Regression		Multivariable Cox Regression	
	**HR (95% CI)**	***P-*Value**	**HR (95% CI)**	***P-*Value**
Age at diagnosis (> 60 *vs* ≤ 60 years)	2.57 (1.66–3.98)	< 0.0001	1.70 (1.07–2.70)	0.024
BMI (> 25 *vs* ≤ 25)	1.73 (1.14–2.61)	0.007	-	NS
Peritoneal carcinomatosis (Yes *vs* No)	2.28 (1.51–3.45)	0.0001	1.87 (1.23–2.85)	0.003
sPD-L1 (> 0.42 *vs* ≤ 0.42 ng/mL)	3.01 (1.85–4.89)	< 0.0001	2.23 (1.34–3.73)	0.002
sPD-1 (> 2.48 *vs* ≤ 2.48 ng/mL)	1.62 (1.04–2.50)	0.02	-	NS
sBTN3A1 (> 4.75 *vs* ≤ 4.75 ng/mL)	2.74 (1.75–4.30)	< 0.0001	-	NS
pan-sBTN3As (> 13.06 *vs* ≤ 13.06 ng/mL)	2.53 (1.63–3.94)	< 0.0001	-	NS
sBTN2A1 (> 5.59 *vs* ≤ 5.59 ng/mL)	1.92 (1.22–3.03)	0.004	-	NS
sBTLA (> 2.78 *vs* ≤ 2.78 ng/mL)	2.18 (1.41–3.36)	0.0002	-	NS

*Abbreviations: BMI* Body Mass Index, *HR* Hazard Ratio, *NS* Not Significant

### Validation cohort

**Fig. 3:**
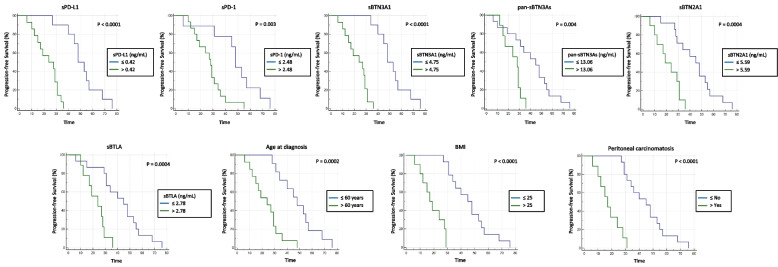
Kaplan–Meier analysis of progression-free survival in twenty-four advanced HGSOC patients from validation cohort. Abbreviation: BMI, Body Mass Index

Lastly, we used a further independent cohort of 24 peripheral blood samples from advanced HGSOC patients in order to confirm the previously observed correlations for each tested parameter. The previously determined concentration thresholds in leading cohort by the ROC curve approach were used to perform a Kaplan–Meier survival analysis. As expected, we observed a significant inverse correlation between PFS and high expression levels in plasma for each biomarker/factor (Fig. 3). This confirms and emphasizes our previous results obtained in the study leading cohort (Fig. 2). Therefore, advanced HGSOC women with plasma levels below the specified thresholds showed a gain in median PFS which was from 17 to 26 months longer than patients with levels above the concentration *cut-offs*.

Additionally, also for women from the validation cohort the impact on PFS of age at diagnosis, baseline BMI and peritoneal carcinomatosis at onset was evaluated by Kaplan–Meier curves. This further analysis showed similar results to those obtained in the leading cohort, suggesting that age at diagnosis > 60 years, BMI > 25, or presence of peritoneal carcinomatosis were associated with a shorter PFS. Finally, multivariate analysis performed on validation cohort confirmed that low plasma PD-L1 levels (≤ 0.42 ng/mL), age at diagnosis ≤ 60 years and absence of peritoneal carcinomatosis are favorable independent prognostic factors for PFS in women with advanced HGSOC (Table S3).

## Discussion

In recent years, interesting results from phase II and III trials for the clinical management of patients affected by several solid tumors were obtained by the blocking of the PD-1/PD-L1 immune regulatory complex [22].

OCs (especially HGSOCs) have been shown to be potentially immunogenic tumors rich in tumor-infiltrating immune cells. The abundance of tumor-infiltrating cellsmodulates the anticancer immune response and provides the optimal condition to develop effective immunotherapy approaches [12, 23]. However clinical studies evaluating the effectiveness of these therapies (PD-1/PD-L1 inhibitors) in OC patients so far did not yield the expected results, showing response rates of < 15% [24].

Over the years, several studies assessed the ability of PD-1 and PD-L1 to act as a biomarkers for tumor prognosis, suggesting that their high expression is associated with poor clinical outcome in patients with different cancer types, including OC [25].

Hamanishi et al. [26] reported that PD-L1 expression by tumor was correlated with decrease in intraepithelial TILs and poor survival in epithelial OC (EOC). Additionally, Wieser and collaborators [25] showed that all OC subtypes, except mucinous, exhibited high PD-1 and PD-L1 expression levels, especially advanced OCs and tumors harboured by younger patients. No difference in PD-1 and PD-L1 expression between HGSOC and low grade serous ovarian cancer (LGSOC) was detected [25]. High PD-1 levels have been shown to be directly associated with more advanced FIGO stages and high tumour grade, while PD-L1 expression was correlated with tumor grade only [25, 27, 28]. Unlike PD-L1 which showed no association with survival [29], instead an increased PD-1 expression was able to predict a poor PFS [25]. Conversely, a recent study [30] highlighted that high tumor PD-L1 expression, determined through immunohistochemistry (IHC), was associated with poor prognosis in LGSOC. However, tumor PD-L1 prognostic value is still debated and has not been fully elucidated in OC [28].

In general, several technical limitations regarding tissue sampling, methodology and used antibodies were found during assessment of PD-L1 expression by IHC analysis in formalin-fixed paraffin-embedded (FFPE) tissue samples. Because PD-L1 and PD-1 are dynamic biomarkers as well as the immune system, assessing their expression in primary tumor tissue may not provide an overview of metastatic disease, which evolves during tumor progression [21].

In the last years, circulating PD-1 and PD-L1 levels have been shown to be associated with worse survival in individuals affected by different cancers [19, 20]. However, the association between increased levels of soluble PD-1 and PD-L1 and poor clinical outcome has been little investigated so far in OC women [31, 32].

Additionally, the soluble forms of other immunomodulatory molecules, such as butyrophilins and BTLA, were tested by our research group in blood from patients affected by several tumors [6, 20, 21].

Constantly scientific research is looking for new prognostic factors which would enable predict patient survival, increasing the effectiveness of therapeutic treatments. There is today little data about immunological predictors in OC. For this purpose, our study focused on analysis of the baseline plasma expression levels of six immunoregulatory molecules, such as sPD-L1, sPD-1, sBTN3A1, pan-sBTN3As, sBTN2A1 and sBTLA, correlating them with survival data from one hundred advanced HGSOC patients. A survival analysis by Kaplan–Meier curves was performed in order to associate the plasma concentrations of these immunomodulatory proteins with PFS of advanced HGSOC patients. This investigation allowed, for each tested circulating biomarker, to discriminate advanced HGSOC patients based on long (≥ 30 months) versus short PFS (< 30 months). We surprisingly observed that circulating levels of each tested soluble protein were negatively associated with PFS in advanced HGSOC patients. Advanced HGSOC patients with plasma levels of tested immune checkpoints below the established concentration threshold showed a median PFS ranging 6 to 16 months longer compared to that of patients with concentrations above the threshold.

Although several previous studies showed that the assessment of tumor PD-L1 may not be a prognostic factor for OC due to its controversial role, our study instead demonstrated that its circulating form is inversely correlated with PFS of advanced HGSOC patients.

An increase in plasma levels of immune checkpoints relatively to their specific concentration thresholds has been shown to be associated with poor prognosis, therefore, these could be used in the future as potential prognostic biomarkers. Our study, for the first time, highlighted that assessing the circulating levels of some immunomodulatory molecules could concur to prognosticate survival of advanced HGSOC patients, allowing to implement optimal therapeutic strategies and discriminate those women who may take advantage from tailored therapies. Hence, the use of the plasma sPD-L1, sPD-1, sBTN3A1, pan-sBTN3As, pan-sBTN3As, sBTN2A1 and sBTLA concentrations as “inspectors” able to monitor clinical outcome of advanced HGSOC patients could be helpful to improve patient clinical management as well as prevent needless healthcare costs.

OC is considered a disease of the elderly as the average age of diagnosis is around 60 years [33]. Several studies also investigated the prognostic impact of age on survival of OC patients, showing controversial results [34–36]. Some authors reported that advanced age was not an independent prognostic factor in OC, but the poor clinical outcome observed in elderly women could be attributed to other associated adverse prognostic factors [37]. Our analysis by Kaplan–Meier curves confirmed these previous observations, showing that advanced HGSOC women with age at diagnosis over 60 years had a lower PFS (< 30 months) than others.

Additionally, our investigation also evaluated the impact of baseline BMI on survival of advanced HGSOC patients, since the incidence of obesity is increasing in the developed world and it is associated with an increased risk of malignancy, contributing to 14%-20% of cancer-related mortality [38]. Previous studies highlighted an association between obesity and poor survival in several tumor types, including OC [39, 40]. Conversely, Skírnisdóttir et al. [41] reported that overweight and obese patients with OC did not shown worse survival than normal weight and underweight patients. Therefore, the correlation between obesity at diagnosis and survival of OC patients still remains controversial [42]. Our findings, instead, suggest a negative effect of excess body weight (BMI > 25) on PFS of advanced HGSOC women. Further studies are needed to elucidate the molecular and hormonal mechanisms underlying these clinical observations.

Other factors such as peritoneal carcinomatosis, which frequently occur in late-stage disease, could affect clinical outcome of OC patients [43, 44]. Our study confirmed that peritoneal carcinomatosis at diagnosis is associated with a lower PFS.

Finally, a multivariate analysis carried out to investigate the impact of different baseline covariates on PFS showed that plasma levels of sPD-L1 ≤ 0.42 ng/mL, age at diagnosis ≤ 60 years and absence of peritoneal carcinomatosis were significant prognostic factors for a longer PFS in advanced HGSOC patients. Therefore, plasma sPD-L1 levels, age at diagnosis and presence/absence of peritoneal carcinomatosis rather than other immune checkpoints or BMI should be considered before starting the therapeutic treatment for advanced HGSOC patients.

Recently, Parvathareddy et al. [45] demonstrated a discordance in the PD-L1 expression between primary EOC and their corresponding sites of peritoneal dissemination, suggesting the association between PD-L1 expression in peritoneal disseminationand adverse prognostic factors, including high histological grade. The patients with advanced stage tumors frequently have ascites which could also be a source of several soluble factors such as sPD-L1. A recent study determined the sPD-L1 levels in peritoneal fluid, suggesting its role as unfavorable prognostic factor in OC [32].

For this reason, the correlation between plasma sPD-L1 levels and peritoneal carcinomatosis with a shorter PFS observed in our study acquires greater emphasis.

Several studies were performed to investigate the dynamics of sPD-L1 variations in patients receiving ICI treatment, showing controversial results depending on the type of tumor [46–48]. In general, low disease control rates were associated with high pretreatment sPD-L1 levels. Therefore, sPD-L1 levels were independent predictors of PFS and OS in patients receiving ICI treatment for advanced tumors [49–51]. In future, assessing sPD-L1 levels could become a strategy to select patients able to respond to the immunotherapies.

Lastly, analyses performed on independent validation cohort of 24 advanced HGSOC women confirmed the previously obtained results.

The innovation of our study was to perform a serial study on plasma, a biological specimen which can be easily isolated, repeatedly, with little invasiveness, and which give us a more dynamic profile of the status of the tumor microenvironment even during therapy, thus overcoming the technical limitations of tissue biopsy (low dynamism, poor quantity of sample and invasiveness). In this analysis, the soluble forms of immune checkpoints were detected in plasma rather than serum, because serum concentrations have been shown to be ten times lower than those detected in plasma from the same blood sample. However, further investigations are needed to discover the release modalities of these soluble forms from tumors.

## Supplementary Information

**Additional file 1: Table S1.** Clinical and pathological characteristics of advanced HGSOC patients. **Table S2.** Characteristics of ELISAs for sPD-L1, sPD-1, pan-sBTN3As, sBTN3A1, sBTN2A1, and sBTLA. **Table S3.** Univariate and multivariate analysis of biomarkers and other factors for PFS in the validation cohort. **Figure S1.** Receiver Operating Characteristic (ROC) curve analysis of age at diagnosis, BMI and plasma levels of PD-L1, PD-1, BTN3A1, pan-BTN3As, BTN2A1, BTLA.

## Data Availability

All data generated or analysed during this study are included in this published article.
