# Supplementary material for: Low plasma PD-L1 levels, early tumor onset and absence of peritoneal carcinomatosis improve prognosis of women with advanced high-grade serous ovarian cancer
Source: BMC Cancer. 2023 May 13;23:437. doi: 10.1186/s12885-023-10911-5 (PMC10183131; doi:10.1186/s12885-023-10911-5)

Supplementary Tables

Table S1. Clinical and pathological characteristics of advanced HGSOC patients.

Characteristic	No. of Patients (%)
**Total patients**	100
**Age at diagnosis (y):**<br>Median: 61<br>Mean: 60<br>Range: 27-79	100
**Age groups (y)**	
≤ 60	48 (48)
> 60	52 (52)
**FIGO stage** [a]	
IIIB	23 (23)
IIIC	52 (52)
IV	25 (25)
**Histological grade**	
G1/2	0 (0)
G3	100 (100)
**Histological subtype**	
Serous	100 (100)
Other	0 (0)
**OC**	
Unilateral	64 (64)
Bilateral	36 (36)
**Surgery**	
Surgical staging	52 (52)
Cytoreductive surgery	48 (48)
**Peritoneal carcinomatosis**	
Yes	43 (43)
No	57 (57)
**BMI**	
≤ 25	59 (59)
> 25	41 (41)
**Smoker**	
Yes	23 (23)
No	77 (77)

[a] AJCC Cancer Staging Manual 8th staging.
Abbreviations: BMI, Body Mass Index; FIGO, International Federation of Gynecology and Obstetrics.

Table S2. Characteristics of ELISAs for sPD-L1, sPD-1, pan-sBTN3As, sBTN3A1, sBTN2A1, and sBTLA.

	PD-L1	PD-1	pan-BTN3As*	BTN3A1*	BTN2A1	BTLA
Coating Ab	α-PD-L1 1.8 + α PD-L1 2.1	α-PD-1 6.4	α-BTN3A S148	α-BTN3A1 S240	α-BTN2A1 8.16	α-BTLA 75.2
Detection Ab (biotinylated)	A-PD-L1 1.3.1	α-PD-1 3.1	α-BTN3A 103.2	α-BTN3A 103.2	α-BTN2A1 4.15 + α-BTN2A1 5.28	α-BTLA 7.1 + α-BTLA 8.2
Detection limit (pg/ml)	20	50	100	100	30	200

* Three isoforms of BTN3A are identified (A1, A2, A3). Among available monoclonal antibodies to BTN3A, one is specific for A1 (α-BTN3A1 S240). Coating with α-BTN3A1 S240 allows specific assay of the A1 isoform, whereas the couple of antibodies α-BTN3A S148 and α-BTN3A 103.2 allows simultaneous detection of all 3 forms (Pan-BTN3A assay). It is however noteworthy that BTN3A concentrations obtained with the Pan-BTN3A assay are only indicative since the range used in the assay is pure BTN3A1. BTN3A concentrations should therefore be expressed as pg/ml « equivalent BTN3A1».

Table S3. Univariate and multivariate analysis of biomarkers and other factors for PFS in the validation cohort.

Factor/biomarker	Univariate Cox Regression		Multivariable Cox Regression	
	HR (95% CI)	*p*-Value	HR (95% CI)	*p*-Value
Age at diagnosis (>60 *vs* ≤60 years)	5.64 (2.03-15.6)	0.0009	8.12 (2.24-29.5)	0.0015
BMI (>25 *vs* ≤25)	33.1 (4.14-265)	0.001	-	NS
Peritoneal carcinomatosis (Yes *vs* No)	12.7 (3.65-44.2)	0.0001	48.9 (6.98-343)	0.0001
sPD-L1 (>0.42 *vs* ≤0.42 ng/mL)	4.30 (1.55-11.9)	0.005	4.47 (1.30-15.3)	0.02
sPD-1 (>2.48 *vs* ≤2.48 ng/mL)	26.5 (3.29-213)	0.0021	-	NS
sBTN3A1 (>4.75 *vs* ≤4.75 ng/mL)	34.1 (4.18-277)	0.001	-	NS
pan-sBTN3As (>13.06 *vs* ≤13.06 ng/mL)	4.30 (1.46-12.7)	0.008	-	NS
sBTN2A1 (>5.59 *vs* ≤5.59 ng/mL)	5.86 (1.93-17.8)	0.002	-	NS
sBTLA (>2.78 *vs* ≤2.78 ng/mL)	6.14 (2.04–18.5)	0.0013	-	NS

Abbreviations: BMI, Body Mass Index; HR, Hazard Ratio; NS, Not Significant.

Figure S1. Receiver Operating Characteristics (ROC) curve analysis of age at diagnosis, BMI and plasma levels of PD-L1, PD-1, BTN3A1, pan-BTN3As, BTN2A1, BTLA.

For each parameter, ROC curves were plotted for sensitivity and specificity of survival classification. The figure shows the optimal values of specificity and sensitivity for optimal threshold values (*Youden index* associated criterion). AUC, Area Under the Curve. $P<0.05$.

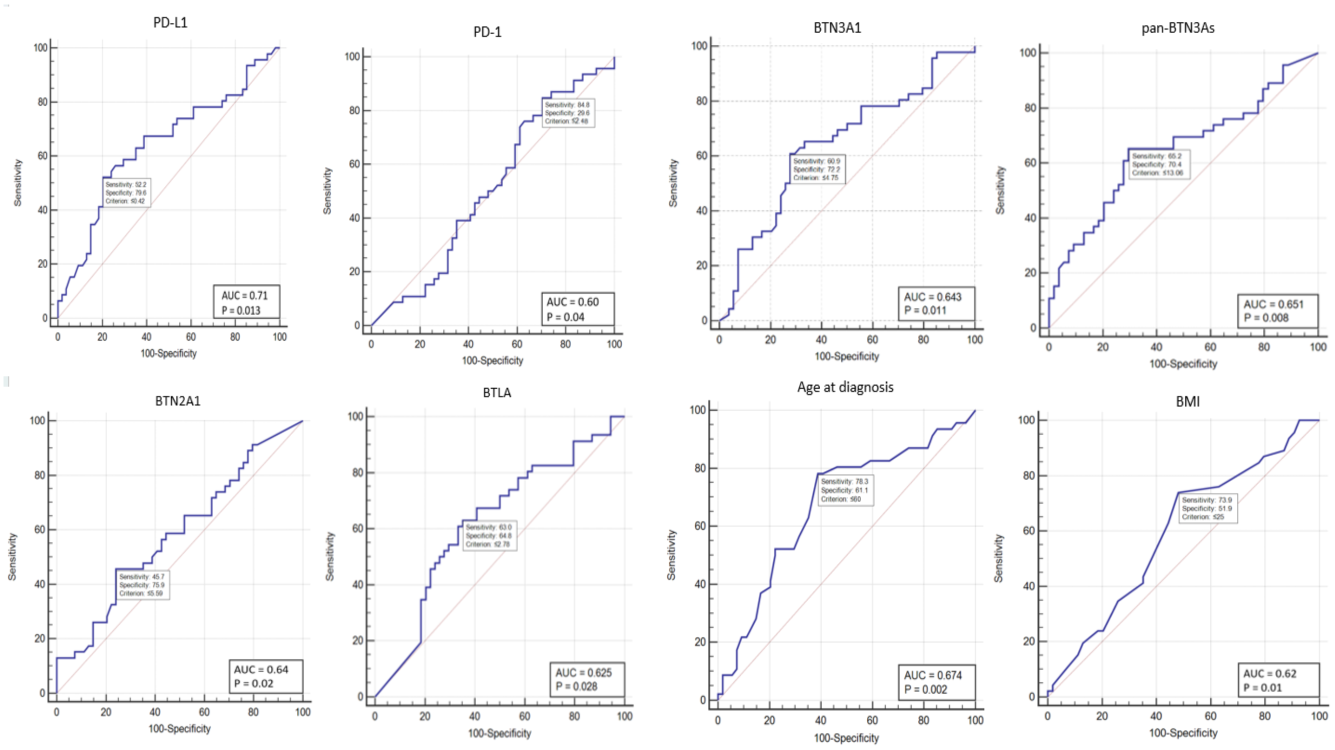

Supplement: Supplementary file 1 — Additional file 1: Table S1. Clinical and pathological characteristics of advanced HGSOC patients. Table S2. Characteristics of ELISAs for sPD-L1, sPD-1, pan-sBTN3As, sBTN3A1, sBTN2A1, and sBTLA. Table S3. Univariate and multivariate analysis of biomarkers and other factors for PFS in the validation cohort. Figure S1. Receiver Operating Characteristic (ROC) curve analysis of age at diagnosis, BMI and plasma levels of PD-L1, PD-1, BTN3A1, pan-BTN3As, BTN2A1, BTLA. [file 12885_2023_10911_MOESM1_ESM.pdf]
